# Advances in Human Immune System Mouse Models for Personalized Treg-Based Immunotherapies

**DOI:** 10.3389/fimmu.2021.643544

**Published:** 2021-02-18

**Authors:** Isabelle Serr, Maria Kral, Martin G. Scherm, Carolin Daniel

**Affiliations:** ^1^Group Immune Tolerance in Type 1 Diabetes, Helmholtz Diabetes Center at Helmholtz Zentrum München, Institute of Diabetes Research, Munich, Germany; ^2^Deutsches Zentrum für Diabetesforschung (DZD), Neuherberg, Germany; ^3^Division of Clinical Pharmacology, Department of Medicine IV, Ludwig-Maximilians-Universität München, Munich, Germany

**Keywords:** HIS mice, Treg, cancer immunotherapy, personalized medicine, iPSC-derived HSCs, microRNA

## Abstract

Immunodeficient mice engrafted with a functional human immune system [Human immune system (HIS) mice] have paved the way to major advances for personalized medicine and translation of immune-based therapies. One prerequisite for advancing personalized medicine is modeling the immune system of individuals or disease groups in a preclinical setting. HIS mice engrafted with peripheral blood mononuclear cells have provided fundamental insights in underlying mechanisms guiding immune activation vs. regulation in several diseases including cancer. However, the development of Graft-vs.-host disease restrains relevant long-term studies in HIS mice. Alternatively, engraftment with hematopoietic stem cells (HSCs) enables mimicking different disease stages, however, low frequencies of HSCs in peripheral blood of adults impede engraftment efficacy. One possibility to overcome those limitations is the use of patient-derived induced pluripotent stem cells (iPSCs) reprogrammed into HSCs, a challenging process which has recently seen major advances. Personalized HIS mice bridge research in mice and human diseases thereby facilitating the translation of immunomodulatory therapies. Regulatory T cells (Tregs) are important mediators of immune suppression and thereby contribute to tumor immune evasion, which has made them a central target for cancer immunotherapies. Importantly, studying Tregs in the human immune system *in vivo* in HIS mice will help to determine requirements for efficient Treg-targeting. In this review article, we discuss advances on personalized HIS models using reprogrammed iPSCs and review the use of HIS mice to study requirements for efficient targeting of human Tregs for personalized cancer immunotherapies.

## Introduction

With the discovery of checkpoint inhibitors and more recently, the use of chimeric antigen-receptor T cells, immunotherapies have taken center stage in oncology. The common goal for cancer immunotherapies is to boost the immune system to attack and destroy cancerous cells. The gold-standard for pre-clinical testing of cancer therapies has long been the use of xenograft models, where human cancer cell lines are engrafted into immunodeficient hosts. However, both development and evaluation of immunotherapies requires the presence of a functional immune system closely mirroring the immune reaction in the human disease. Immunodeficient mice engrafted with a functional human immune system [human immune system (HIS) mice] have opened up new ways of evaluating immunotherapies directly *in vivo* in a pre-clinical setting. One main obstacle remaining in cancer therapy is intra-tumor heterogeneity. The use of personalized medicine offers a promising approach to tackle this major hurdle and HIS mice engrafted with patient-derived hematopoietic cells are an important tool for the development of personalized medicine.

## Human Immune System Mouse Models for Immunotherapies

Since the discovery that severe combined immunodeficiency (*Prkcd*^*scid*^ or SCID) mice, engrafted with human hematopoietic stem cells (HSCs) or human peripheral blood mononuclear cells (PBMCs), can develop a human immune system (1), researchers are constantly working on improving xenoengraftment in such HIS mice to study human diseases. Advancements have been made especially regarding background strain, immunodeficiency mutation as well as engraftment method and material, thereby considerably improving engraftment efficacy (2). For example, mice on the NOD background present with the highest engraftment efficacy due to a polymorphism in the gene *Sirpa*, encoding the signal regulatory protein alpha (*Sirpa*) that shows enhanced interaction with human CD47 on hematopoietic cells, thereby preventing their phagocytosis by macrophages (3).

Moreover, to overcome human T cell xeno-reactivity directed against murine MHC molecules, strains of immunodeficient mice lacking murine MHC class I and class II were developed (4, 5). We have used murine MHC class II deficient NOD-*scid* IL2Rgamma^null^ (NSG)-HLA-DQ8 transgenic mice engrafted with fresh human cord blood (CB) HSCs and demonstrated a high engraftment efficacy in peripheral blood of these animals (6). Importantly, we identified autoreactive disease-relevant insulin-specific CD4^+^T cells, indicating positive selection on HLA-DQ8 molecules in the thymus of humanized mice (6). The development of T cells with a TCR repertoire recapitulating that of the human donor is essential to analyze T cell responses, also in the setting of cancer immunotherapy. In this regard, in a mouse model for prostate adenocarcinoma, tumor-infiltrating regulatory T cells (Tregs) showed a significant enrichment for distinct TCR specificities targeting an antigen present in healthy pancreatic tissue and not specific to the tumor (7). Therefore, the use of HIS mice with transgenic expression of human HLA molecules will be important to permit for accurate representation of TCR repertoires to study T cell responses to the tumor.

In order to study tumor related immune responses in the human immune system *in vivo*, HIS mice engrafted with cell line-derived (CDX), or patient-derived xenografted tumors (PDXs) have been established (8, 9). Importantly, MART1-TCR-transgenic T cells from humanized mice reconstituted with fetal thymic tissue and human HSCs were able to mount efficient HLA/antigen-dependent anti-melanoma immune responses also after transfer into tumor-bearing recipients (10). Among others, especially the potent checkpoint inhibition with anti-PD1 humanized antibodies has been tested in HIS mice bearing various types of tumors (8, 9, 11), highlighting the value of PDX engrafted HIS models for cancer immunotherapies.

## HIS Mice as Preclinical Model for the Treatment of Cancer

Despite the therapeutic advances indicated above, some hurdles remain which hinder efficient and safe implementation of cancer immunotherapies to the patient. During disease progression, genomic instability leads to genetic variations within tumor cells resulting in various distinct populations of cancer cells (12). This intra-tumor heterogeneity is a major obstacle for the success of efficient immunotherapies since it fosters resistance to therapy and leads to highly variable treatment outcomes thereby calling for personalized strategies.

The potential of personalized HIS mouse models for the analysis of patient specific immune responses was demonstrated in a PDX-bearing HIS mouse model based on NSG mice engrafted with autologous tumor-infiltrating T cells and tumor cells from the same patient. Importantly, the autologous T cell transfer was successful in eradicating tumors only in mice generated with material from patients that show a positive autologous cell transfer response in the clinic (13).

To investigate complex immune responses more closely in a preclinical personalized setting, the engraftment of HIS mice with autologous hematopoietic cells and tumor cells from the same patient is required. This can be achieved by engraftment with patient-derived PBMCs. However, such mice are not useful for long-term studies as they can develop severe Graft-vs.-Host-Disease (GvHD) within few weeks upon engraftment. Alternatives for long-term studies are the engraftment with patient-derived HSCs or HSCs originating from fetal liver or CB. Here, engraftment with patient-derived HSCs allows for positive selection of T and B cells based on murine antigens in the thymus and bone marrow, therefore limiting GvHD reactions and further enabling MHC compatibility with the autologous tumor tissue. One drawback of HSC engraftment, however, are low frequencies of HSCs in the peripheral blood of adults without mobilization, which impede an effective reconstitution of the human immune system in HIS mice. Of note, in 2006, a group led by Shinya Yamanaka generated pluripotent stem cells from adult cells. The discovery of these induced pluripotent stem cells (iPSCs) has thenceforward substantially revolutionized the field of translational research and personalized medicine (14) and enabled an unlimited source for the derivation of HSCs (15, 16).

## Next Generation HIS Mouse Models Using iPSC-Derived HSC for Personalized Medicine

**Figure 1:**
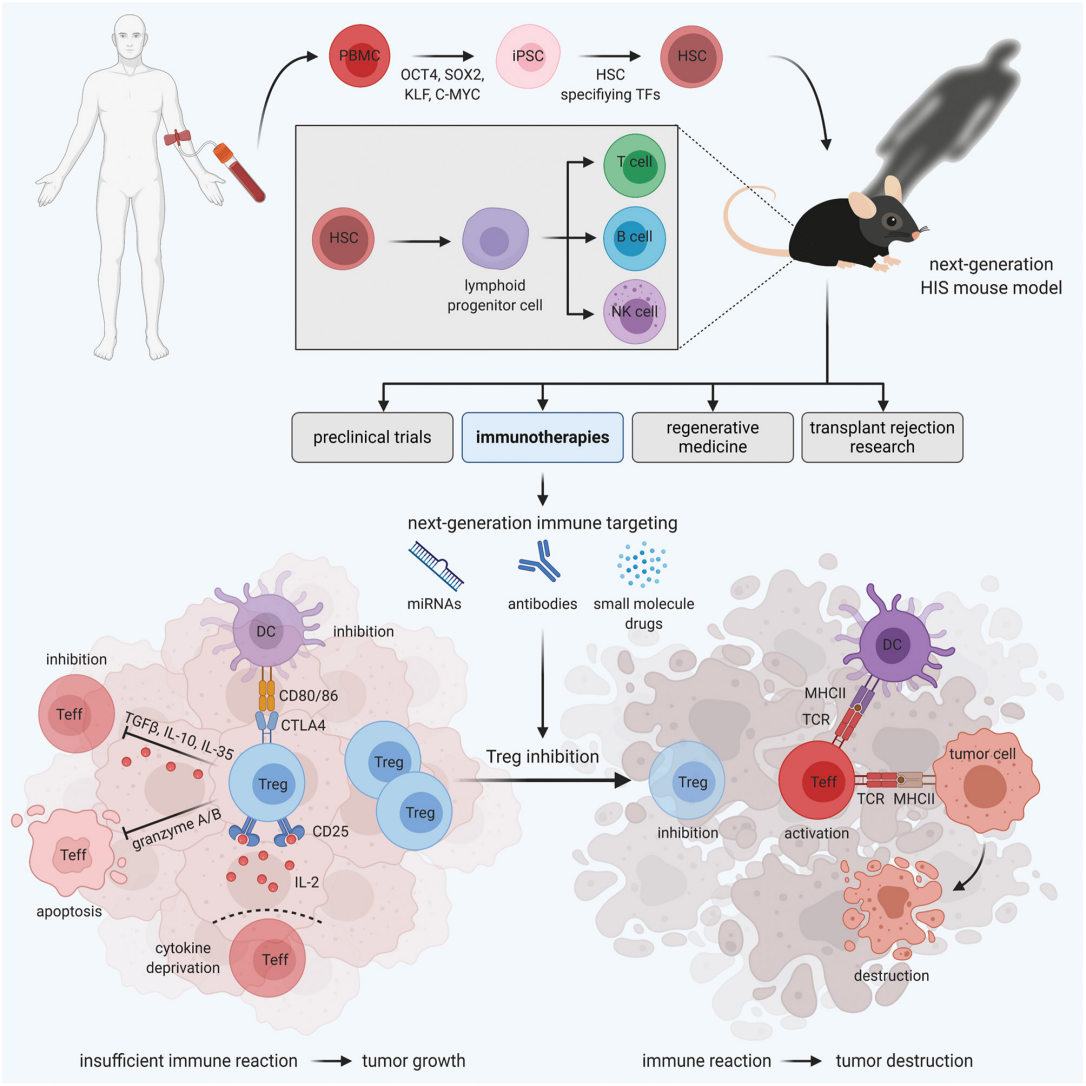
HIS mouse models for personalized Treg-based cancer immunotherapies. Immunodeficient mice engrafted with human PBMC-derived HSCs develop a functional human immune system. These HIS mice are valuable tools for various areas of medical research, including preclinical trials, regenerative medicine, transplant rejection research, and immunotherapies. In the field of immunotherapies, next-generation HIS mouse models enable the development and testing of novel immune targeting strategies, aiming at Treg inhibition to boost the immune reaction against the tumor, in a human immune system *in vivo*.

The discovery that iPSCs are able to self-renew and differentiate into any cell type by introducing the reprogramming factors Oct4, Sox2, cMyc, and Klf4 (14, 17) has enabled the development of novel therapeutic strategies in personalized and regenerative medicine. Noteworthy, iPSCs can be derived from different sources of somatic cells, thereby evading the limitations of using primary, patient-derived disease affected cells for e.g., disease modeling (18) (Figure 1).

Yet, *de novo* generation of functional HSCs from iPSCs for efficient *in vivo* engraftment and multi-lineage potential remains challenging due to the complex nature of hematopoietic ontogeny (19). Recently, methods for the generation of self-renewing hematopoietic stem and progenitor cells with multi-lineage potential after engraftment into immunodeficient hosts have been developed. Although analysis was largely limited to the bone marrow, T and B cells, as well as myeloid cells were also detected in spleen, thymus and peripheral blood, showing a diverse TCR repertoire and functional antibody responses (16, 20). These initial studies highlight the potential use for the generation of HIS mice with iPSC-derived HSCs (20). Furthermore, hPSCs generated from iPSCs from patients with Diamond-Blackfan anemia (DBA) were used to enable a new therapeutic pathway for the treatment of DBA (21) and immunodeficient mice reconstituted with patient-derived iPSCs were used to develop a drug-testing system for chronic myelomonocytic leukemia (22). Even though still low in number, such studies highlight the vast opportunities and potential therapeutic applications that are offered by iPSC technology, which have to be further explored and improved for future investigations.

Personalized HIS mice reconstituted with tumor cells and autologous patient-derived hematopoietic cells from the same donor are especially important, since they will help to mirror the immunological tumor microenvironment. Importantly, the tumor microenvironment contains immunosuppressive immune subsets [reviewed in (23)], including Tregs that promote tumor immune evasion and hence impede effective antitumor immune responses [reviewed in (24)].

## Targeting Tregs for Cancer Immunotherapies in HIS Mice

Tregs are a specialized suppressive subtype of CD4^+^T cells that controls the immune response in the periphery and prevents adverse immune reactions and autoimmunity (25). They are characterized by the high expression of the high-affinity interleukin-2 receptor alpha-chain (CD25) and the expression of the transcription factor Foxp3, which is the master regulator of their phenotype and function (26–28). Mutations in the *Foxp3* gene lead to defects in Treg development and function as illustrated by severe multiorgan autoimmunity in patients with the Immunodysregulation polyendocrinopathy enteropathy X-linked (IPEX) syndrome (29) and mice with Scurfy mutations (30). While defects in Treg function and reduced frequencies are associated with autoimmune disorders, increased Treg infiltration into solid tumors and the resulting suppression of effector immune cells are associated with poor prognosis in several types of cancer (31–35). Accordingly, the manipulation of Tregs has gained interest in the field of cancer immunotherapy. However, many aspects of Treg-mediated immune suppression in tumors remain to be determined. Importantly, HIS mice reconstituted with CB HSCs or PBMCs display comparable frequencies of Tregs in various tissues as non-humanized mice (6, 11, 36–40), making them a suitable tool to study human Tregs *in vivo* in a preclinical model (Figure 1). Notably, the development of immune cells in NSG mice is skewed to the lymphoid lineage, with cells of the myeloid lineage being under represented, due to limited cross-reactivity of murine cytokines with human hematopoietic cells [reviewed in (41)]. Therefore, novel humanized mouse strains such as NSG-SGM3 mice (42) and others (43) that transgenically express human cytokines and thereby support myeloid cell development have been developed. Importantly, it was demonstrated that NSG-SGM3 mice further support the development of Foxp3^+^Tregs (44), thereby highlighting the importance of considering different HIS mouse models as basis for pre-clinical studies.

It is now established, that Tregs take residence in various tissues, where they express tissue specific signature genes and exert key non-canonical functions (45–49). Notably, a tissue specific signature has been likewise identified for tumor-infiltrating Tregs. Importantly, the investigation of gene expression of Tregs isolated from breast cancer, colorectal cancer, non-small cell lung cancer and hepatocellular carcinoma in comparison to Tregs from adjacent healthy tissue or peripheral blood identified a tumor-specific Treg gene expression signature (50–52). Some of these signature genes (e.g., *CTLA4, GITR, CCR4*) are likewise present on peripheral Tregs, whereas their expression is increased in tumor-infiltrating Tregs. Other tumor-specific Treg signature genes (e.g., *CCR8, FCRL3, IL1R2*) are exclusively expressed on tumor-infiltrating Tregs and absent in their peripheral counterparts. However, the functional consequence of this gene signature in tumor-infiltrating Tregs remains to be determined.

Checkpoint inhibitors have taken center stage in cancer immunotherapy research, since they block signaling through immune inhibitory molecules, especially programmed cell death protein 1 (PD-1) and cytotoxic T-lymphocyte associated protein 4 (CTLA4) (53, 54). Many tumors promote immune evasion by expression of PD-L1, a ligand for PD-1, which is in turn expressed on activated T cells. The engagement of PD-1 by PD-L1 is highly immunosuppressive and leads to T cell anergy and apoptosis. Accordingly, blocking PD-1/PD-L1 signaling has shown great efficacy in various cancers, however in a large proportion of patients PD-1/PD-L1 blockade shows limited success, highlighting the importance of understanding underlying mechanisms of checkpoint inhibitor blockade as well as defining biomarkers to predict treatment success (39). In addition to effector T cells, PD-1 is likewise expressed on Tregs and multiple studies indicate, that PD-1 blockade can increase Treg proliferation and suppression, which could account for the differences in treatment outcome (53). In this regard, the ratio of PD-1^+^CD8^+^T cells/PD-1^+^Tregs in the tumor microenvironment can predict the efficacy of PD-1 blocking therapy (39). While PD-1 blockade has been studied extensively in HIS mice, only few studies investigated the specific effect on Tregs. In line with highly variable treatment outcomes in patients, two studies did not observe changes in tumor-infiltrating Treg frequencies (11, 38), while one study using a combination of anti-PD-1 antibody and CD137 antibody therapies reported an increase in CD8/Treg ratio in tumors of HIS mice xenotransplanted with gastric carcinoma and engrafted with autologous PBMCs from the same donor (55). These studies highlight the strength of personalized HIS mice using xenotransplanted tumors and autologous immune cells for the assessment of treatment outcome.

The identification of tumor-related Treg signature genes is an important step toward more specific targeting of tumor Tregs without affecting the peripheral Treg pool which could potentially lead to autoimmune reactions. CTLA4 mediated tolerization of antigen-presenting cells is an important mechanism of suppression by Tregs. Importantly, CTLA4 blockade has been studied extensively as checkpoint inhibitor in cancer immunotherapy, since CTLA4 is often upregulated by tumor cells for immune evasion (54). However, the anti-tumor effects of anti-CTLA4 treatment largely rely on the antibody-dependent cellular cytotoxicity (ADCC) mediated depletion of tumor-infiltrating Tregs (56, 57). Here, HIS mice offer an important advantage over classical non-humanized mouse models in the pre-clinical assessment of depleting antibodies. ADCC relies on the interaction of the human IgG Fc region on depleting antibodies with human immune cells and can therefore be studied accurately only in the presence of these cells (58, 59). Anti-CTLA4 antibodies were used to successfully deplete Tregs in fetal liver engrafted HIS mice treated with low-dose IL2 to demonstrate that critical side effects and toxicity of high-dose IL2 treatment are largely dependent on the depletion of Tregs (40). This study highlights the usefulness of HIS mice to study not only treatment efficacy, but also critical side effects of cancer immunotherapy.

Like CTLA4 the chemokine receptor type 4 (CCR4), albeit being expressed on peripheral Tregs, is further upregulated on tumor-infiltrating Tregs, while additionally being expressed on different types of cancer cells. To test anti-CCR4 treatment in a pre-clinical setting, a humanized mouse model for lymphoma based on CCR4^+^ lymphoma bearing NOG mice reconstituted with human PBMCs was used (60). The treatment with an anti-CCR4 antibody induced robust ADCC leading to reduced tumor mass, accompanied by decreased Treg frequencies in the tumor (60). The latter finding suggests that anti-CCR4 treatment might be more generally applicable also to CCR4-negative tumors, by targeting tumor-infiltrating Tregs. Accordingly, a Phase Ia clinical trial demonstrated efficient Treg depletion by an anti-CCR4 antibody in patients with CCR4-negative solid tumors resulting in stable disease in half of the patients 12 weeks after treatment start (61). However, CCR4 is likewise expressed on peripheral Tregs, albeit at lower levels, leading to the simultaneous depletion of peripheral Tregs by anti-CCR4 treatment. Tumor cells and cells in the tumor microenvironment secrete high amounts of CCL22, a ligand for CCR4, in various cancers (32, 62, 63). Therefore, small molecule CCR4 antagonists might be a safer alternative to block Treg migration to the tumor microenvironment without Treg depletion.

An alternative to Treg depletion is the manipulation of their functional properties. In this regard, glucocorticoid-induced tumor necrosis factor receptor-related protein (GITR) has been studied extensively as a target for cancer immunotherapies. GITR is a coreceptor, which is expressed at low levels on naïve CD4 and CD8 T cells and is upregulated upon activation with Foxp3^+^Tregs harboring the highest levels of GITR (64, 65). Importantly, targeting GITR for cancer immunotherapy became of interest with the discovery that an agonist anti-GITR antibody could break self-tolerance in mice by making effector T cells resistant to Treg suppression (66). Accordingly, anti-GITR antibody treatment could induce strong anti-tumor immunity in mice (67–69). While these studies demonstrated that the anti-GITR antibody acts mainly by enhancing effector T cell frequencies through avoidance of Treg mediated suppression, the exact mechanism remains unclear. In addition to rendering effector T cells resistant to Treg mediated suppression, agonist anti-GITR treatment was suggested to likewise enhance the effector T cell: Treg ratio in the tumor (67, 70). This change was attributed to the agonist anti-GITR antibody rendering Tregs unstable, causing them to lose Foxp3 expression (67), or to Treg depletion (70) in different studies. In NSG mice reconstituted with human hCD34^+^HSCs and grafted with melanoma tumors the treatment increased the effector T cell: Treg ratio in the spleen and the tumor, while remaining tumor-infiltrating Tregs showed reduced expression of activation markers such as ICOS (70). Overall, although the treatment was not able to clear the tumor completely, treated mice exhibited significantly reduced tumor growth (70). The exact mechanisms leading to the enhanced effector T cell: Treg ratio in this particular study remain to be defined. The loss of activation markers such as ICOS observed in the anti-GITR study in HIS mice could indicate that, in addition to Treg depletion, remaining Tregs are rendered phenotypically unstable by the treatment.

Destabilization of Tregs is a promising approach for Treg-based immunotherapy and mechanisms of Treg destabilization have been studied in the setting of autoimmune diseases. Treg stability is mainly mediated by epigenetic mechanisms, most importantly the demethylation of the conserved non-coding sequence 2 (CNS2) in the *Foxp3* locus (71). We were able to show that microRNA (miRNA) 142-3p contributes to Treg instability during progression of Type 1 Diabetes (T1D) by targeting TET2, a molecule that can actively demethylate DNA (37, 72, 73). MiRNAs are small non-coding RNAs that can sequence-specifically inhibit their target mRNAs, thereby regulating complex cellular states, such as T cell activation, which makes them important targets for immunotherapy (74). Importantly, miRNA modulation has been studied extensively in the setting of autoimmunity and infection. Accordingly, a miR122 inhibitor is currently being tested in clinical trials for hepatitis C virus (HCV) infections, highlighting the feasibility of targeting miRNAs for immune modulation in human diseases (75). Regarding miRNA modulation for Treg targeting, we used NSG mice reconstituted with PBMCs and were able to demonstrate that the blockade of miRNAs that impact Treg induction or stability *in vivo* enhances human Treg frequencies both in the periphery and in the pancreas (36, 37, 76). Lessons learned from the autoimmune setting could be used for cancer immunotherapy. Enhancing the function of these miRNAs using miRNA mimics could induce immune responses against cancer cells by reducing Treg-mediated suppression mechanisms. Importantly, miRNAs that induce autoimmunity when overexpressed in lymphocytes, can still have tumor-promoting properties because of their expression in other cell types (e.g., miR17~92 cluster) (77, 78). Therefore, the implementation of miRNA modulation for immune targeting in cancer therapy relies on the development of targeted approaches that allow the modulation of these miRNAs specifically in immune cells or even specific subsets. Importantly, miRNA delivery systems are currently intensively studied [reviewed in (79)], as miRNA instability and degradation still hinder the development of a successful system. To improve the delivery of miRNAs in a preclinical setting that also enables the testing of drug safety and efficiency, HIS mouse models offer a suitable platform prior the translation to humans.

## Outlook and Conclusions

The perpetual improvements of HIS mouse models have enabled the design of personalized immunotherapies for various diseases including cancer and the evaluation of their efficacy prior to the translation to the human setting. Using patient-specific HIS mice will enable us to better understand the heterogeneity of immune responses and advance the development of novel personalized immunotherapies for the treatment of cancer. Even though still in its infancy, the use of iPSC-derived HSCs for engraftment could be an important step toward personalized HIS models.

Additionally, the immunosuppressive tumor microenvironment influences antitumor responses and the efficiency of cancer immunotherapies. Targeting Tregs has therefore become an important tool in cancer immunotherapy and has been studied extensively in murine models. In order to target Tregs most efficiently in human diseases the development of novel models depicting the immune reaction of the human disease is pivotal. HIS mouse models offer the opportunity to study the interaction of human immune cells, including Tregs, with human tumors directly *in vivo*.

Overall, current advances in establishing a “truly” humanized mouse model for the development of immunotherapies and drug delivery systems will guide personalized medicine approaches further to the translation to the clinic.

## Author Contributions

IS and MK reviewed the literature and wrote the manuscript. MGS reviewed the manuscript and prepared the figure using BioRender. CD reviewed the literature and contributed to the conceptualization of the manuscript. All authors contributed to the article and approved the submitted version.

## Conflict of Interest

The authors declare that the research was conducted in the absence of any commercial or financial relationships that could be construed as a potential conflict of interest.
